# Expression of *acrA* and *acrB* Genes in *Esherichia coli* Mutants with or without *marR* or *acrR* Mutations

**Published:** 2013-12

**Authors:** Razieh Pourahmad Jaktaji, Nasim Jazayeri

**Affiliations:** 1 Department of Genetics, Faculty of Science and Biotechnology Center, University of Shahrekord, Shahrekord, Iran; 2 students in Genetics at University of Shahrekord

**Keywords:** *acrAB* operon, *acrR*, *marR*, Multiple antibiotic resistance

## Abstract

***Objective(s):*** The major antibiotic efflux pump of *Esherichia coli* is AcrAB-TolC. The first part of the pump, AcrAB, is encoded by *acrAB* operon. The expression of this operon can be kept elevated by overexpression of an activator, MarA following inactivation of MarR and AcrR repressors due to mutation in encoding genes, *marR* and *acrR*, respectively. The aims of this research were to use *E. coli* mutants with or without mutation in *marR* to search for the presence of possible mutation in *acrR* and to quantify the expression of *acrAB*.

***Materials and Methods:*** The DNA binding region of *acrR* gene in these mutants were amplified by PCR and sequenced. The relative expression of *acrA* and *acrB* were determined by real time PCR.

***Results:*** Results showed that W26 and C14 had the same mutation in *acrR*, but none of the mutants overexpressed *acrA* and *acrB* in comparison with wild type strain.

***Conclusions: ***The effect of *marR* or *acrR* mutation on *acrAB* overexpression is dependent on levels of resistance to tetracycline and ciprofloxacin.

## Introduction

Generation of multiple drug resistant phenotypes of pathogenic bacteria, like *Esherichia coli* is a worldwide clinical concern. These phenotypes are associated with increase in the activity of membrane transporters mainly AcrAB-TolC, which belongs to the resistance-nodulation-division (RND) family of transporters (1). This transporter or efflux pump consists of three ingredients, including AcrA, a periplasmic membrane-fusion protein; AcrB, the inner membrane protein; and TolC, an outer membrane channel. These ingredients are encoded by *acrA*, *acrB* and *tolC*. The first two genes are located in the same operon, while *tolC* is placed on different site of bacterial chromosome (2, 3). 

**Figure 1:**
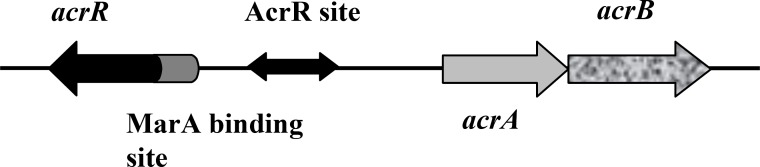
*acrAB* operon, *acrR* and the regulatory region between them. Modified and adapted from Dzwokai *et al* (12)

AcrR, the repressor of *acrAB* operon is encoded by *acrR* gene (4). Its location is upstream of *acrAB* operon and transcribed divergently from the same promoter (Figure 1). Attachment of AcrR through its DNA-binding helix-turn-helix (HTH) motif to operator site of *acrAB* operon causes operon repression (6). On the other hand, this operon is under the positive regulation of MarA, a transcriptional activator (7). Its binding site is shown in Figure 1. 

Expression of MarA happens following dissociation of MarR repressor from operator site of *marRAB* operon (8). Both AcrR and MarR repressors possess DNA binding and drug binding sites (6, 9). In separate studies, it was shown that mutation in their encoding genes, *marR* and *acrR*, can maintain the overactivity of the AcrAB-TolC pump (10, 11). It was found that two clones C14 (without a mutation in *marR*) and C16 (with a mutation in *marR*) slightly overexpressed *marA* (12, 13). This in turn may promote overexpression of *acrAB* operon. The aims of this research were first, to study the possible presence of mutations in *acrR* gene and then to quantify the expression of *acrA* and *acrB* in these clones. 

## Materials and Methods

***Antimicrobial agent and media***

 The stock of 4 mg/ml tetracycline hydrochloride (Tc) (Sigma) was used in this research. LB broth (Merck, Germany) and LBA containing 1.5% agar (Merck, Germany) were used for cultivation of control strain and mutants. 

**Table 1:** Bacterial strain and mutants

Strain/Mutant/Clone	Relevant properties	MIC		Source/Reference
		Ciprofloxacin (ng/ml)	Tetracycline (µg/ml)	
MG1655	Wild type	35	3	A gift from Prof. R. G. Lloyd
W26	Wild type; *gyrA* (Ser_83_→Leu)	75	4	Pourahmad & Mohiti, 2010
W49	Wild type; *gyrA* and *marOR* (20 bp duplication in operator)	625	4	Pourahmad & Mohiti, 2010
C14	W26; *gyrA* (Ser_83_→Leu)	1000	30	Pourahmad & Ebadi, 2013
C16	W49; *gyrA* and *marOR* (20 bp duplication in operator)	1000	30	Pourahmad & Ebadi, 2013

***Bacterial strain and mutants***

Bacterial strain and mutants are listed and described in Table 1. MG1655 was the wild type strain. W26 and W49 are mutants isolated from cultivation of wild type strain on LBA plus 40 ng/ml ciprofloxacin (14). Clones C14 and C16 were generated during the previous work (15). They were derived from mutants W26 and W49 following cultivation on LBA agar containing up to 20 µg/ml Tc (15). Based on the previous data, these clones and mutants show low to medium levels of resistance to ciprofloxacin and tetracycline (16, 17). 

***PCR amplification and DNA sequencing of acrR***

 PCR was used to amplify the 5′ end of *acrR* gene in wild type and mutants (14). A single colony from each mutant and clone on LB agar was suspended in 100 µl of sterile water and after boiling at 95˚C for 3 min; it was cooled on ice and used as a PCR template for *acrR* amplification. Forward and reverse primers for amplification were 5΄-CACGAACATATGGCACG-3΄ and 5΄-GCCTGATACTCAAGCTC-3΄, respectively. The amplified PCR products were 240 bp. The sequence of these products was compared with that of MG1655 obtained from NCBI (NC_000913.2) following DNA sequencing. 

***acrA and acrB expression analysis by real time PCR***

 A fresh culture of bacteria was prepared in LB broth plus 3 µg/ml Tc (except for the wild type) and incubated at 37°C with shaking at 150 rpm and grown to mid-logarithmic phase. Each culture was used for extraction of RNA using an RNeasy Mini Kit (Qiagen, Germany) following stabilization in RNA protect bacterial reagent (Qiagen. Germany). RNase-free DNase I was used to eliminate contaminating genomic DNA according to the manufacturer's instruction (Fermentas, Life science research) and the absence of DNA was confirmed by amplification of RNA samples plus a DNA sample as a positive control. The concentration of total RNA was estimated at OD_260_ using spectrophotometer (Ultrospec 1100, Amersham Pharmacia Biothech). 

Purified total RNA (2 µg) was used as a template in RT-PCR using a RevertAid Reverse Transcriptase kit (Fermentas, Life science research). The cDNAs obtained from reverse transcription were used to quantify the level of *acrA*, *acrB* and *gapA*, as an endogenous reference gene by real time PCR in a Rotor Gene 6000 thermocycler (Corbett Research, Australia) using a SYBR Green kit (Takara, Japan). The specific primers used for real time PCR are listed in Table 2. Thermal cycling conditions were described previously (3). Relative gene expression was calculated using the efficiency method pfaffl (ratio of target gene expression, *acrA* and *acrB*, to *gapA* expression) (18). All data on gene expression are the mean of triplicate analyses. The data were presented as mean±SD. Statistical analysis of relative expression was done by SPSS version 16. T-test was used for comparison of relative gene expression data.

## Results

**Figure 2:**
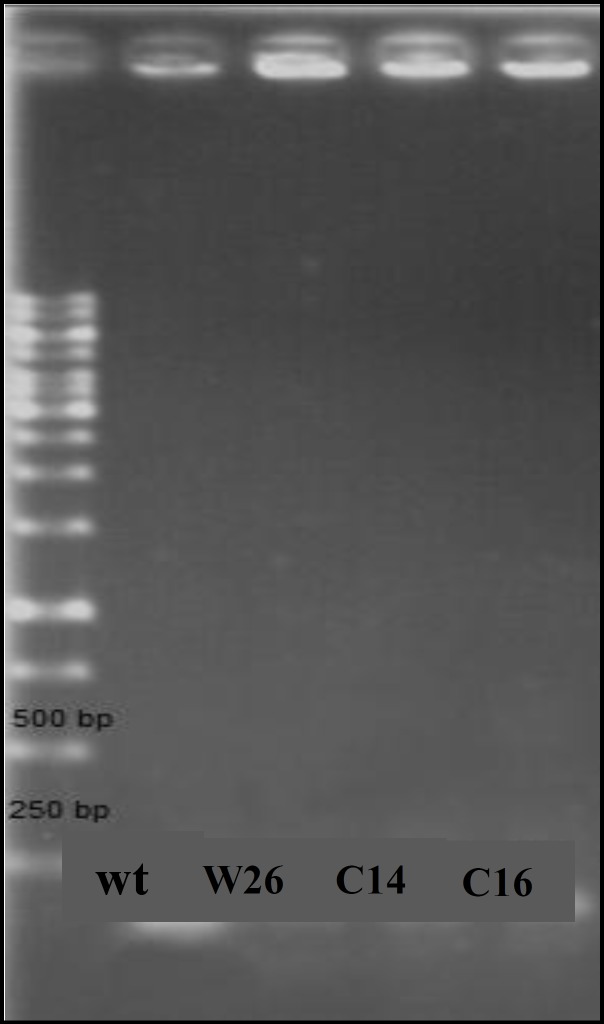
PCR products of *acrR* gene in wild type (wt) and mutants. First lane shows the 1 Kb ladder and other lanes show PCR products

**Figure 3:**
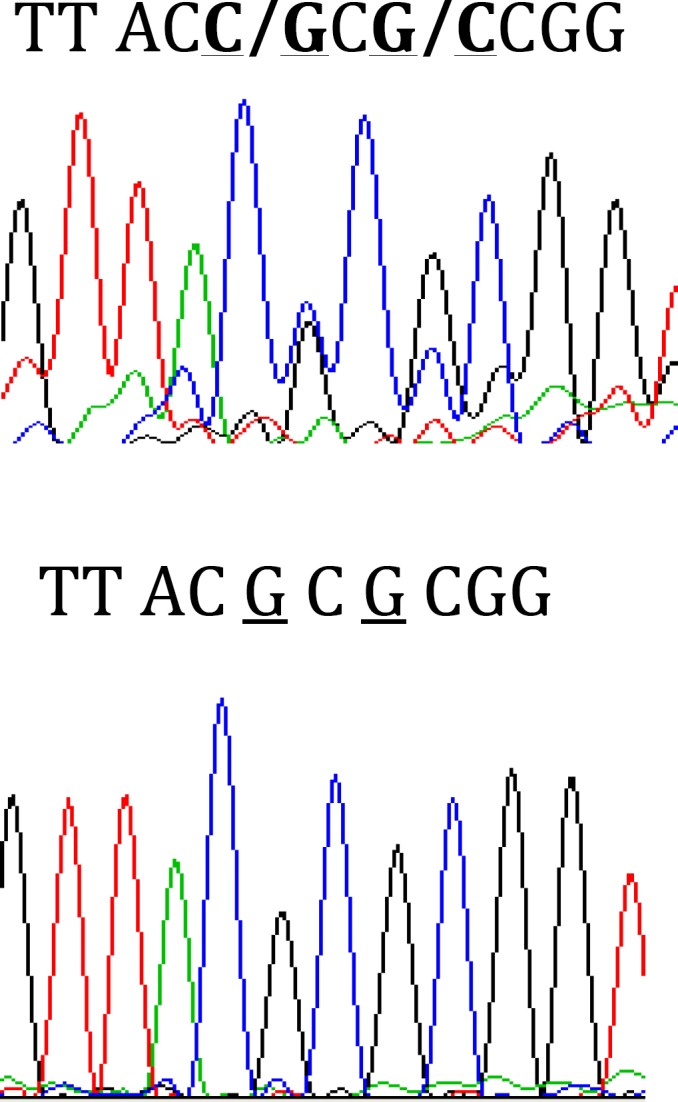
Sequence output from *acrR* PCR product of C14 mutant (first part) and wild type (second part) using forward and reverse primers. Underlined nucleotides show the differences between nucleotide sequences of two parts

 Mutants were used to be evaluated for the presence of possible mutation in 5΄ end of *acrR* gene corresponding to HTH motif of encoded protein and for quantification of *acrAB* expression. Figure 2 shows the result of gel electrophoresis of the *acrR* PCR product of MG1655 and mutants. The comparison of nucleotide sequence of PCR products following DNA sequencing with published sequence of *acrR* in MG1655 showed that W26 and C14 had the same changes in *acrR*. Figure 3 shows the comparison of nucleotide sequence of C14 PCR product with that of the wild type. However, other mutants and clones were the same as the wild type. Thus, all mutants and clones had just a single mutation either in *marR* or *acrR*.

**Table 2:** List of real time PCR primers

Gene	Primer sequence (5′-3′)	Length of amplicon (bp)	Reference
*acrA*	F: TTGAAATTACGCTTCAGGAT	189	Viveiros *et al*, 2007
	R: CTTAGCCCTAACAGGATGTG		
*acrB*	F: CGTACACAGAAAGTGCTCAA	183	Viveiros *et al*, 2007
	R: CGCTTCAACTTTGTTTTCTT		
*gapA*	F: ACTTACGAGCAGATCAAAGC	170	Viveiros *et al*, 2007
	R: AGTTTCACGAAGTTGTCGTT		

A G/C heterozygote genotype at nucleotide position 131 in coding region of *acrR* in W26 and C14 would not change amino acid Thr at codon 44 and a G/C heterozygote genotype at position 133 could change Arg (CGC) to Pro (CCC) at codon 45. Substitution of Arg-45 with Cys, but not with Pro was reported previously (10). 

Real time PCR results reveal that the efficiency of *acrA*, *acrB* and *gapA* were 1.96, 1.99 and 2.1, respectively. The melting curve of two genes showed just one major peak which indicates the purity of samples. The melting point of three genes was 86-88^o^C. Table 3 shows the *acrA* and *acrB* relative expression in wild type and others. The t-test analysis showed no significant difference among the expression of these genes in the wild type and in mutants and clones (*P*<0.05). This shows the low induction of *acrAB* promoter in these bacteria. 

Furthermore, as *acrA* and *acrB* are in the same operon, it was expected that both of them show almost the same result for the level of expression.

## Discussion

The increased level of resistance to fluroroquinolones, such as ciprofloxacin and other structurally unrelated antibiotics, like tetracycline which causes multiple resistance phenotypes is attributed to over activation of multidrug efflux pumps, mainly AcrAB-TolC pump in *E. coli* (1-3). Overactivity of this pump was seen even in other bacteria following the induction with increasing amounts of tetracycline or acquiring high levels of resistance (19).

Generally, fluroroquinolone resistance has been attributed to point mutations in the quinolone resistance-determining regions of the target genes, such as *gyrA* (20). However, higher levels of resistance can be achieved following overactivation of AcrAB-TolC pump (21). This happens following the overexpression of *marA *and thereby overactivation of *acrAB* and *tolC* genes. The expression of these genes has been determined in clinical isolates by real time PCR (22-24). Therefore, it was decided to quantify the expression of *acrA* and *acrB* genes in mutants and clones with or without a mutation in *marR* after evaluation of the possibility of acquiring mutation in *acrR*. 

**Table 3:** Relative expression of *acrA* and *acrB* in wild type (MG1655) and mutants as determined by real time PCR

Strain/mutant	Relative expression of *acrA*	Relative expression of *acrB*
Wild type (MG1655)	1±0	1±0
W26	1.16±0.021	1.13±0.012
W49	1.62±0.01	1.45±0.015
C14	1.28±0.013	1.17±0.011
C16	1.4±0.013	1.31±0.02

It was found that none of the mutants overexpresses *acrA* and *acrB* following the addition of 3 µg/ml Tc. This may be due to the levels of resistance to antibiotics in mutants and clones. This is also possible that the level of *marA* overexpression in clones was not enough to overexpress *acrAB*. This possibility arises following the suggestion that the level of *marA* overexpression is important for activation of *acrAB* operon (25). 

It was shown that Arg at position 45 of AcrR is highly conserved and its alteration to cystein enhances the expression of *acrB* in mutants with high levels of resistance to ciprofloxacin (10). In the present work, it was found that W26 and its derived clone C14 had alteration at position 45. However, this alteration did not promote overexpression of *acrAB*. This may imply that mutation at this location is not the only cause of *acrAB* overexpression. This is consistent with the previous findings indicating that in stress conditions, expression of *acrAB* enhances independent of AcrR activity. However, after overexpression of *acrAB*, the presence of active AcrR is important to regulate the levels of *acrAB* expression (26). 

Moreover, the importance of the repressor binding site on DNA, and repressor DNA binding motif for MarR and AcrR repressor were mentioned previously as mutations in these locations along with overactivity of MarA promote overexpression of *acrAB* operon (10, 11). The finding that mutants and clones harboring mutations in either of these locations in *marR* or *acrR* could not promote overexpression of *acrA* and *acrB*, again reveals that the level of resistance is important for overexpression of *acrAB* operon. 

In addition, it was shown that tolerance of organic solvants, such as cyclohexane happens following overexpression of *acrAB-tolC* (27). It was previously found that none of these mutants and clones could tolerate cyclohexane (13, 15). Thus, the findings of this study reconfirm the relation between organicsolvent tolerance and the level of *acrAB* expression. 

## Conclusion

 Upregulation of *acrAB* operon occurs after acquisition of high levels of resistance to Tc and ciprofloxacin. Thus, the effect of *marR* or *acrR* mutation on *acrAB* overexpression is dependent on the levels of resistance to these antibiotics. At high levels of resistance, evaluation of the synthesis of AcrAB-TolC pump ingredients along with mRNA quantification by real time PCR would reconfirm AcrAB-TolC overactivity. 
